# Stimulating the Dorsolateral Prefrontal Cortex Decreases the Asset Bubble: A tDCS Study

**DOI:** 10.3389/fpsyg.2019.01031

**Published:** 2019-05-09

**Authors:** Xuejun Jin, Cheng Chen, Xue Zhou, Xiaolan Yang

**Affiliations:** ^1^College of Economics, Zhejiang University, Hangzhou, China; ^2^School of Business and Management, Shanghai International Studies University, Shanghai, China; ^3^Academy of Financial Research, Zhejiang University, Hangzhou, China

**Keywords:** asset bubble, cognitive ability, learning-to-forecast, dorsolateral prefrontal cortex, transcranial direct current stimulation

## Abstract

Many studies have discussed the neural basis of asset bubbles. They found that the dorsolateral prefrontal cortex (DLPFC) played an important role in bubble formation, but whether a causal relationship exists and the mechanism of the effect of the DLPFC on bubbles remains unsettled. Using transcranial direct current stimulation (tDCS), we modulated the activity of the DLPFC and investigated the causal relationship between the DLPFC and the asset bubble in the classical learning-to-forecast experiment. 126 subjects were randomly divided into three groups and received different stimulations (left anodal/right cathodal, right anodal/left cathodal, or sham stimulation), respectively. We also conducted a 2-back task before and after stimulation to measure changes in subjects’ cognitive abilities and explore in detail the cognitive mechanism of the effect of DLPFC stimulation on asset bubbles. Based on our results, we found that the bubble of the left anodal/right cathodal stimulation group was significantly smaller than that of the sham stimulation group. In the meantime, subjects performed significantly better in the 2-back task after left anodal/right cathodal stimulation but not right anodal/left cathodal or sham stimulation, which is consistent with their performance in the learning-to-forecast experiment, supporting the cognitive mechanism to some extent. Furthermore, we examined different forecasting rules across individuals and discovered that the left anodal/right cathodal stimulation group preferred the adaptive learning rule, while the sham and right anodal/left cathodal stimulation groups adopted a pure trend-following rule that tended to intensify market volatility aggressively.

## Introduction

The research on asset bubbles can be traced back to the middle of the last century, but there are still many questions to be settled in this field. To date, volumes of studies have indicated that asset bubbles exist in the real financial market. However, the “Efficient Market Hypothesis” (EMH) proposed by Fama (1970) dominated the mainstream literature in the beginning, claiming that the asset price reflected all available information without deflection, which was proven to be contradicted by the real financial market in later empirical studies (Fischer et al., 1972; Basu, 1977). Such studies have demonstrated through empirical data that the asset price often deviated from its fundamental price by discounting the expected value of future dividends and have defined this phenomenon as the asset bubble. They tried to solve this problem through innovations of economic systems, but none has managed to eliminate bubbles completely (Chancellor, 1999).

With the development of behavioral finance, increasingly more academics have come to the realization that the mechanisms underlying the emergence of bubbles may not be inherent to economic systems but lie in human’s bounded-rational trading behavior under certain conditions (Ogawa et al., 2014). Thereafter, many behavior economists tried to study economic bubble behaviors in a virtual financial market through laboratory experiments, starting with Smith et al. (1988). Many observed large positive bubbles followed by dramatic crashes toward the end of the experiment (Smith et al., 1988; Noussair et al., 2001; Haruvy and Noussair, 2006; Haruvy et al., 2014; Eckel and Fuüllbrunn, 2015). Among these laboratory experiments regarding asset bubbles, “learning-to-forecast” is a typical one introduced by Marimon et al. (1993). In this type of experiment, subjects participated as professional financial advisers and continuously predicted the price of a pension fund based on its dividend and market interest rate. In addition to the large deviation from the fundamental price frequently observed in other experiments, subjects without rational expectations were found to adopt a trend-following strategy during forecasting (Hommes et al., 2005), which tended to increase the volatility of the asset price and induce an asset bubble.

Some neuroimaging studies have explored the neural basis of economic bubble behavior and identified the activation of the dorsolateral prefrontal cortex (DLPFC) during bubbles. They found that the DLPFC participated in decision-making during bubbles in a virtual stock exchange and was associated with asset preference that might bias the decision (Ogawa et al., 2014; Ye et al., 2016). Evidence from a field experiment also confirmed the correlation of DLPFC activation with direct access trading in a real stock market. They found that the success of the trading activity, based on a large number of filled transactions, was related to higher activation of the DLPFC (Raggetti et al., 2017). These findings suggested a correlation between DLPFC activity and economic bubble behaviors, but whether a causal relationship exists remains unsettled. Our main contribution is to confirm the neural basis of the DLPFC on asset bubble behavior using transcranial direct current stimulation (tDCS), one of the brain stimulation techniques.

Moreover, we are interested in the mechanism of the relationship between the DLPFC and bubbles, particularly the cognitive abilities that have been frequently mentioned to affect bubbles by many studies. For example, some researchers who performed learning-to-forecast experiments have pointed out that the observed bubbles in the simplest economic environment may be attributed to subjects’ lack of related knowledge or failing to fully understand the experimental market (Bao et al., 2017). In fact, some experimental studies have managed to reduce or even diminish bubbles by illustrating the whole experiment in a simple and understandable way (Teo, 2011; Huber and Kirchler, 2012; Kirchler et al., 2012; Yamamoto, 2015; Yamamoto et al., 2015). Others found that markets that consisted of subjects with higher cognitive abilities performed better in price efficiency (Bosch-Rosa et al., 2017). Prices in such markets exhibited a more stable pattern and converged closer and more quickly to the rational expectation equilibrium (Zong et al., 2017). Neuroscientific studies have shown that anodal tDCS over the left DLPFC enhances cognitive functioning, especially in working memory (Nitsche et al., 2008; Hoy and Fitzgerald, 2010; Zaehle et al., 2011; Jacobson et al., 2012; Meinzer et al., 2012; Hoy et al., 2013; Dedoncker et al., 2016). Working memory is essentially the capacity to keep information in mind for a short period of time (Kuhnen and Knutson, 2005; Silvanto et al., 2008; Hoy et al., 2013; He et al., 2016; Lerner et al., 2016). Since improvements in working memory have been proven to enhance more complex thought and action through the ability to manipulate information (Jaušovec and Jaušovec, 2012), it is believed to be the most crucial foundation of cognitive processing, and improvements in working memory can represent a considerable enhancement in cognitive ability. Based on previous literature on asset bubbles, we found that working memory capacity (WMC) was one of the most related factors that influenced the asset bubbles in the learning-to-forecast experiment. Intuitively, working memory plays an important role in the prediction of asset price because people need to process the information before forecasting. Indeed, some researchers had provided evidence on the relationship between working memory and prediction. They found that people with higher WMC predicted more accurately (Bröder et al., 2010; Sewell and Lewandowsky, 2012; McDaniel et al., 2014). People with higher WMC tended to maintain and refresh information more actively. They learned from previous data and abstracted systematic regularities to improve their prediction algorithms and hence increased their accuracy of prediction (McDaniel et al., 2014). Furthermore, people with higher WMC predicted more accurately when price movement was approximately non-linear, since they were able to use well-calibrated strategies (Fischer and Holt, 2017). However, lower WMC participants usually adopted simple example-based prediction strategies. In conclusion, previous literature indicated that working memory was important in prediction. Thus, we focused on the influence of WMC in a learning-to-forecast experiment in our paper. Although different studies draw different conclusions on cathodal simulation, most studies have agreed on the positive effect of anodal stimulation on working memory performance measured by either response time or accuracy or both. Therefore, it is reasonable to examine the cognitive mechanism of decreasing bubbles through tDCS over the DLPFC.

Motivated by the above literature regarding neurophysiological traits, asset bubbles and cognitive abilities, we compared subjects’ economic bubble behavior in a classical learning-to-forecast experiment following left anodal/right cathodal, right anodal/left cathodal and sham stimulation by a tDCS device, in an attempt to identify the neural mechanism of their decisions, which tend to induce asset bubbles. In particular, we also collected their performance measures in a working memory task pre and post stimulation to further explore the role of cognition in this process. Combining these two results, we then discuss the causal relationship between asset bubbles and cognitive abilities.

## Materials and Methods

### Participants and Procedure

**Figure 1:**
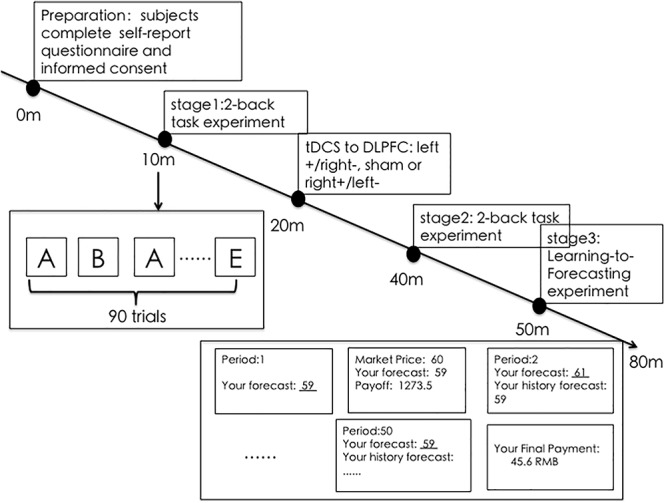
The procedure and schedule of the whole experiment. The subjects were first asked to perform a 2-back task experiment and then randomly separated into different tDCS groups: left+/right–, sham, or right+/left–. After receiving tDCS to the DLPFC, they performed the 2-back task again. Finally, they were asked to perform a 50-period learning-to-forecast experiment.

A total of 126 participants were recruited from Zhejiang University. All of them were asked to complete a self-report questionnaire that included gender, age, major and whether they had participated in a similar experiment before. Each participant was provided written informed consent. This study was carried out in accordance with the recommendations of the Zhejiang University ethics committee. The protocol was approved by the Zhejiang University ethics committee. None of the subjects reported any adverse side effects regarding pain on the scalp or headaches after the experiment. The whole experiment was conducted over the course of several stages. In stage 1, all participants were required to perform a 3-min 2-back task before tDCS. Then, they were randomly allocated to receive 20 min of left anodal/right cathodal (42 subjects), right anodal/left cathodal (42 subjects), or sham (42 subjects) tDCS. Then in stage 2, after the stimulation, they were required to complete a 3-min 2-back task again with a different random letter series. In stage 3, every 6 subjects completed a 50-period learning-to-forecast experiment in an experimental market, generally lasting 30–40 min. The whole procedure and schedule were showed in Figure 1. Subjects were asked to take seats randomly. During the session they were separated by partitions, so they could not observe the decisions of other subjects in the same session. The software we used in this experiment was z-Tree (Fischbacher, 2007) which was widely used in experimental researches.

Our subjects included 73 males and 53 females. Their average age was about 22 years old and 12.6% of them were in majors which might be related to the decision tasks of our experiment, e.g., economic, finance, business, and psychology. 33.4% of them were undergraduates and others were graduates. The detail information about the numbers of subjects by treatment, gender, major, and student type was shown in Table 1.

**Table 1:** Number of subjects.

Stimulation type	Male	Female	Related majors^∗^	Other majors	Under-graduate	Graduate
Anode	30	12	7	35	15	27
Sham	23	19	4	38	12	30
Cathode	20	22	5	37	14	28
Total	73	63	16	110	41	85

^∗^*Economics, finance, business, and psychology*.

### Transcranial Direct Current Stimulation (tDCS)

**Figure 2:**
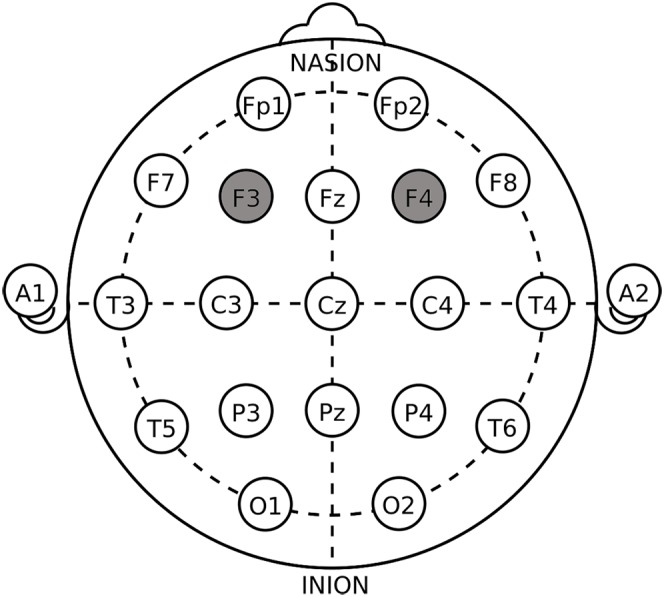
Electrode placement in dorsolateral prefrontal cortex (DLPFC) stimulations.

Transcranial direct current stimulation is a non-invasive form of brain stimulation that has been shown to induce changes in brain activity and subsequent function. tDCS applies a very weak electrical current via two surface electrodes (35 cm^2^) to the scalp, modulating the activity of neurons and therefore influencing subjects’ decision-making process. Anodal stimulation has been shown to depolarize neurons, leading to an increase in brain activity, while cathodal stimulation hyperpolarizes neurons and generally results in a reduction in brain activity. In this study, we used a tDCS device to modulate the subjects’ neural activity in the encephalic region. Based on previous studies, the DLPFC plays an important role in cognitive processing and the formation of asset bubbles. Therefore, we set up three simulation groups: (1) left anodal/right cathodal; (2) right anodal/left cathodal; (3) sham stimulation^1^. The anodal stimulation means that the anodal electrode was placed on the left/right DLPFC (F3/F4 site of the EEG system), while the cathode was on the opposite side of DLPFC (F4/F3 site of the EEG system) (Little and Woollacott, 2014; Little et al., 2014). The EEG system we selected was shown in Figure 2. Active tDCS was delivered for 20 min at 2 mA with a 30-s ramp up and ramp down of current. For the sham stimulation, the current was delivered for only 30 s once it reached 2 mA with the identical 30-s ramp up/ramp down at the beginning and the end of the stimulation.

### Two-Back Task

We chose an adaptive dual 2-back task to identify the effect of tDCS on working memory (Hoy et al., 2013; Martin et al., 2014). In this task, participants would see a series of random letters presented consecutively on the screen for 3 min. They needed to remember the order of the letters and respond by pressing the SPACE button when the present letter was the same as the letter presented two trials earlier. The 2-back task as a whole consists of 90 trials containing approximately 25% targets. Each letter was presented for 2 s in a trial. All participants were asked to perform a 2-back task before and immediately after stimulation, and the order of stimulus presentations was exactly the same across different groups.

### Learning-to-Forecast Experiment

In this phase, we introduced the design of our prime experiment. Our experimental design was mainly based on the classical learning-to-forecast experiment (Hommes et al., 2005). Our experimental parameters were based mainly on the experimental design of Bao et al. (2017) and Zong et al. (2017). The experimental economy was based on a simple asset market with a constant fundamental price. There were six subjects in each market, and each subject played the role of financial adviser in a company. All subjects were told the same information about the asset (dividend = 3.3 yuan and risk-free interest rate = 5%). Each subject’s task was to forecast the asset price in every period, and their experimental rewards only depended on their accuracy of prediction. The whole experiment lasted 50 rounds. In each period, historical prices and their payoffs were displayed to the subjects for reference. Once each of the subjects finished predicting, the realized price based on their predictions would be calculated and made public.

Each period, subjects acted as forecasting advisers and were asked to perform a one-period ahead price predictions $P_{i,t+1}^{e}$. The experimental rewards was totally based upon their own prediction errors in every period as follows:

$$Payoff_{i,t+1}\;=\;\max\;\{0,\left[1300\;-\;\frac{1300}{49}\;{\left(P_{i,t+1}^{e}\;-\;P_{t+1}\right)}^{2}\right]\}\;\;\;\;\;\;\;\;\;\;\;\;\;\;\;\;(1)$$

where $P_{i,t+1}^{e}$ is the prediction of the price at period *t+1* from subject *i* in period *t* and *P*_*t*+1_ is the realized market price at period *t+1*.

The subjects’ predictions were automatically transformed into excess demand for the asset, yielding the following law of motion:

$$P_{t+1}\;=\;66\;+\;\frac{20}{21}\left({\bar{P}}_{t+1}^{e}\;-\;P^{f}\right)\;+\;{\text{ε}}_{t}\;\;\;\;\;\;\;\;\;\;\;\;\;\;\;\;\;\;\;\;\;\;\;\;\;\;\;\;\;\;\;\;\;\;\;\;\;\;\;\;\;\;\;\;\;\;\;\;\;\;\;\;\;\;\;\;\;\;\;\;\;\;\;\;\;\;\;\;\;\;\;\;(2)$$

where *P^f^* = 66 is the fundamental price of the asset, ${\bar{P}}_{t+1}^{e}=$ $\frac{\sum_{i=1}^{6}P_{i,t+1}^{e}}{6}$ is the average prediction price of the six subjects and ε ∼ N(0,1) is a small independent and identically distributed shock to the price *P*_*t*+1_.

With the above price adjustment rule, the subjects’ rewards were maximized if their predictions were all consistent with the fundamental price of 66.

Then, we compared bubbles between different stimulation groups using *t*-tests. To study bubbles from a different perspective, we also examined whether subjects made biased predictions about future asset prices. We followed Haruvy et al. (2007) and estimated the regression model below:

$$P_{t}\;-\;P_{t-1}\;=\;\text{α}\;+\;\text{β}\;\left(P_{t}^{e}\;-\;P_{t-1}\right)\;\;\;\;\;\;\;\;\;\;\;\;\;\;\;\;\;\;\;\;\;\;\;\;\;\;\;\;\;\;\;\;\;\;\;\;\;\;\;\;\;\;\;\;\;\;\;\;\;\;\;\;\;\;\;\;\;\;\;\;\;\;\;\;\;\;\;\;\;\;\;\;\;\;\;\;\;\;(3)$$

This model can be interpreted as follows: if α = 0 and β = 1, then the prediction of price changes is unbiased. Otherwise, the prediction is biased. If β < 1, subjects overpredicted the price. If β > 1, subjects underpredicted the price. The absolute value of β measured the degree of prediction bias.

Third, we focused on the cognitive mechanism involved in 2-back task performances. We calculated and compared the accuracy of the 2-back task before and after stimulation to investigate whether tDCS over the DLPFC successfully modulated subjects’ cognitive performance. The accuracy was calculated by dividing the number of correct responses made by the subject by the total number of targets, shown as follows:

$$\text{Accuracy}\;=\;\frac{Number\;of\;Correct\;Responses}{Number\;of\;Targets}\;\times \;100\%\;\;\;\;\;\;\;\;\;\;\;\;\;\;\;\;\;\;\;\;\;\;\;\;\;\;\;\;\;\;\;\;\;\;\;\;\;\;\;\;\;\;\;\;\;(4)$$

Finally, we investigated the behavioral rules of subjects while predicting asset price. According to Heemeijer et al. (2009), in a learning-to-forecast experiment, subjects mainly adopted two typical rules to forecast asset price: adaptive expectations and trend-following rules. These two classical forecast rules can be shown as follows:

$$\text{Adaptive}\;\text{expectation}\;\text{rule}:\;\;P_{t}^{e}\;=\;P_{t-1}^{e}\;+\;\text{φ}\;\left(P_{t-1}\;-\;P_{t-1}^{e}\right)\;\;\;\;\;\;\;\;\;\;\;\;\;\;\;(5)$$

$$\text{Trend-following}\;\text{rule}:\;\;P_{t}^{e}\;=\;P_{t-1}\;+\;\text{φ}\;\left(P_{t-1}\;-\;P_{t-2}\right)\;\;\;\;\;\;\;\;\;\;\;\;\;\;\;\;\;\;\;\;\;\;\;\;\;\;\;(6)$$

The above two forecasting rules can be described as the following linear formula with α = 1 and β = 0 representing the pure trend-following rule and α + β = 1 representing the adaptive expectation rule.

$$P_{t}^{e}\;=\;\text{α}\;\times \;P_{t-1}\;+\;\text{β}\;\times \;P_{t-1}^{e}\;+\;\text{γ}\;\left(P_{t-1}\;-\;P_{t-2}\right)\;\;\;\;\;\;\;\;\;\;\;\;\;\;\;\;\;\;\;\;\;\;\;\;\;\;\;\;\;\;\;\;\;\;\;\;\;\;\;\;\;\;\;(7)$$

The trend-following rule uses an anchor that gives all weight to the last observed price, while the adaptive expectation rule takes the last forecast into consideration as well, revealing a more aggressive attitude of the former and a more cautious attitude of the latter (Bao et al., 2017).

### Data Analysis

All subjects tolerated the tDCS well, and no adverse effects were reported. First, we analyzed the effects of tDCS on bubbles. To compare asset bubbles across different treatments, we quantified the asset bubble following the standard bubble measurement (Porter and Smith, 1995; Stockl et al., 2010) relative absolute deviation (RAD), defined as the relative distance between realized prices and the fundamental price, as shown below:

$$RAD\;=\;\frac{\left|P_{t}\;-\;P^{f}\right|}{P^{f}}\;\times \;100\%\;\;\;\;\;\;\;\;\;\;\;\;\;\;\;\;\;\;\;\;\;\;\;\;\;\;\;\;\;\;\;\;\;\;\;\;\;\;\;\;\;\;\;\;\;\;\;\;\;\;\;\;\;\;\;\;\;\;\;\;\;\;\;\;\;\;\;\;\;\;\;\;\;\;\;\;\;\;\;\;\;\;\;\;\;\;\;\;\;\;\;\;\;\;\;\;\;\;\;\;\;\;\;\;\;(8)$$

where *P_t_* and *P^f^* denote the market price and fundamental price in period *t*, respectively.

Statistical analysis was performed using Stata statistical software (version 14.0). Statistical description, one-way analysis of variance (one-way ANOVA) and panel regression were used in statistical analysis^2^.

## Results

### Effect of tDCS on Bubbles

**Figure 3:**
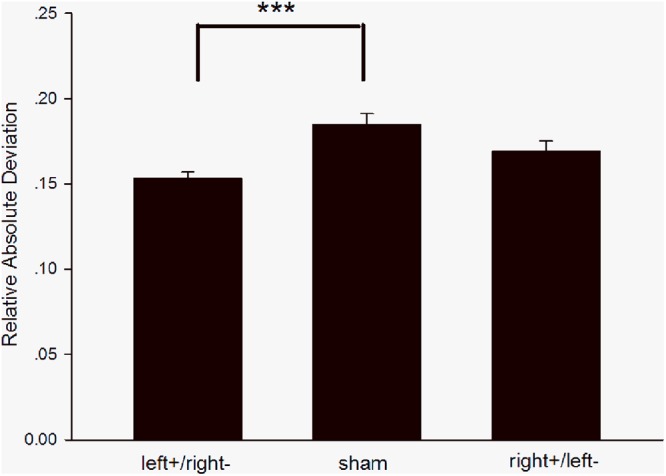
Relative absolute deviation in different groups. The RAD of the left+/right– group was significantly smaller than that of the sham group, while there was no significant difference between the right+/left– group and the sham group. ^∗∗∗^*p* < 0.001, Error bars indicated 95% confidence intervals.

There were significant differences in RAD between the left anodal/right cathodal stimulation group and the sham stimulation group [*F*_(1,698)_ = 13.47, *p* = 0.0003, ES = 0.138; shown in Figure 3]. Markets consisting of subjects receiving left anodal/right cathodal stimulation had smaller bubbles than markets consisting of subjects receiving sham stimulation. We also found that bubbles were not significantly reduced after subjects received right anodal/left cathodal stimulation [*F*_(1,698)_ = 3.27, *p* = 0.0709, ES = 0.068]. Therefore, we concluded that left anodal/right cathodal tDCS over the DLPFC significantly reduced market bubbles, whereas right anodal/left cathodal or sham tDCS to the DLPFC had no significant effect on market bubbles.

There was still an interesting question we wanted to study regarding whether subjects made biased predictions about future market behavior, i.e., whether they consistently over- or underpredicted prices. To answer this question, we estimated equation (3).

According to the results of the regression in Table 2, we found that all groups (left anodal/right cathodal, sham, and right anodal/left cathodal) made biased predictions and overestimated the changes in market price (β < 1). In addition, the sham stimulation group (β = 0.021) and right anodal/left cathodal stimulation group (β = 0.022) overestimated the price changes more than the left anodal/right cathodal group (β = 0.573), consistent with larger bubbles in the sham and right anodal/left cathodal stimulation groups. The fact that all groups did not make unbiased predictions explained why asset bubbles generally existed in all groups.

**Table 2:** Regression on price change.

*price_t_ - price*_*t*-1_	(1) left +/right -	(2) sham	(3) right +/left -
*forecast_t_ - price*_*t*-1_	0.573***	0.021***	0.022***
	(0.0703)	(0.0026)	(0.003)
constant	0.184***	0.356***	0.285***
	(0.028)	(0.0466)	(0.045)

*Standard errors in parentheses. ^∗∗∗^*p* < 0.01*.

### Effect of tDCS on the 2-Back Task

**Figure 4:**
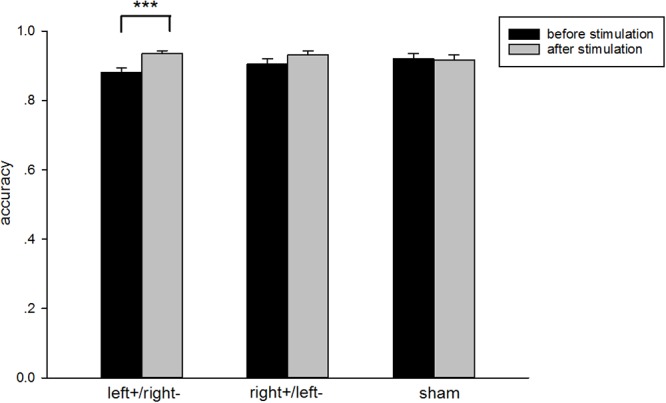
Accuracy in the 2-back task. Black bars, pre-transcranial direct stimulation (pre-tDCS); gray bars, post-tDCS. The subjects’ accuracy in the 2-back task showed a significant increase in the left+/right– group and no significant differences in the right+/left– or sham groups. ^∗∗∗^*p* < 0.001, Error bars indicated 95% confidence intervals.

There was no significant difference in subjects’ accuracy of 2-back task [one-way ANOVA: *F*_(2,99)_ = 2.89, p = 0.0603, ES = 0.169] before they received stimulation. There was a significant increase in 2-back task accuracy after subjects received left anodal/right cathodal tDCS to the DLPFC [one-way ANOVA: *F*_(1,74)_ = 11.13, *p* = 0.0013, ES = 0.3878; shown in Figure 4]. However, we did not find significant differences in 2-back task accuracy after subjects received right anodal/left cathodal or sham tDCS to the DLPFC [one-way ANOVA: *F*_(1,62)_ = 0.32, *p* = 0.5733, ES = 0.07; *F*_(1,62)_ = 1.89, *p* = 0.1745, ES = 0.17, respectively]. The results of our experiment were consistent with previous studies (Hoy et al., 2013; Sellers et al., 2014; Plewnia, 2017; Wang et al., 2018). Therefore, anodal stimulation to the left DLPFC indeed increased subjects’ cognitive abilities.

**Figure 5:**
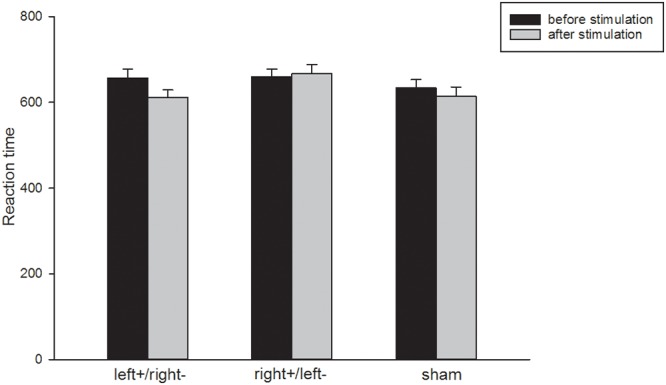
Reaction time in the 2-back task. Black bars, pre-transcranial direct stimulation (pre-tDCS); gray bars, post-tDCS. The subjects’ reaction time in the 2-back task had no significant difference after receiving stimulation. Error bars indicated 95% confidence intervals.

There was no significant difference in subjects’ reaction time of the 2-back task [one-way ANOVA: *F*_(2,99)_ = 0.50, *p* = 0.6069, ES = 0.070; shown in Figure 5] before they received stimulation. The decreasing in reaction time after receiving left anodal/right cathodal tDCS to the DLPFC to the DLPFC was also not significant [one-way ANOVA: *F*_(1,74)_ = 2.77, *p* = 0.105, ES = 0.191]. In fact, it was difficult to capture the change of reaction time, since the reaction time was so short (2 s).

In summary, the results of the 2-back task showed that left anodal/right cathodal stimulation to DLPFC indeed improved the subjects’ cognitive abilities.

### Correlation Analysis on 2-Back Task and Bubbles

To provide evidence to show that the formation of market bubbles was related to the cognitive ability of subjects, we analyzed the relationship between the average 2-back accuracy of subjects in one market and the bubbles created by their trading. As discussed previously, RAD was used to measure bubbles. Using the aggregating data for the experimental markets, we found that the correlation coefficient between the average 2-back accuracy and RAD was -0.332 (Pearson correlation analysis, *p* = 0.179).

We try another way to investigate the relationship between subjects’ cognitive abilities and asset bubbles in the markets. Firstly, we ranked all markets by subjects’ average accuracy in the 2-back task and divided them into two groups, namely the high cognition group and the low cognition group. Then, we compared bubbles in these two groups. The results showed that the RAD of the high cognition group was significantly smaller than that of the low cognition group [one-way ANOVA, *F*(1,898) = 9.08, *p* = 0.0027]. It indicated that on average, markets with subjects who performed better in the 2-back task created fewer bubbles. Thus, there was a negative relationship between subjects’ cognitive abilities and asset bubbles. Combined with our previous results on the effects of tDCS, our findings implied that stimulating the DLPFC decreased the asset bubble and the main reason could be attributed to the increase of cognitive ability.

### Behavior Rules

In this section, we show the differences in the behavioral rules and discuss the relationship between behavioral rules and asset bubbles. To further examine subjects’ behavioral rules for predicting asset price, we estimated equation (7).

$$P_{t}^{e}\;=\;\text{α}\;\times \;P_{t-1}\;+\;\text{β}\;\times \;P_{t-1}^{e}\;+\;\text{γ}\left(P_{t-1}\;-\;P_{t-2}\right)\;\;\;\;\;\;\;\;\;\;\;\;\;\;\;\;\;\;(7)$$

From the regression results in Table 3, we found that the coefficient of the former forecasting price was not significantly different than zero for the sham and right anodal/left cathodal group. The coefficient of the trend (1.138 for sham; 0.748 for right anodal) was large in the sham and right anodal/left cathodal group. In summary, the sham stimulation group was more exposed to pure trend-following behavioral rules, which tended to expand the volatility of market prices and finally caused larger market bubbles (Bao et al., 2017; Bosch-Rosa et al., 2017). Indeed, the bubbles of the sham stimulation group were much larger because they only paid attention to the trend in price without adjusting their expectations. The results of the right anodal/left cathodal group were close to the sham group. The left anodal/right cathodal group took not only the trend but also their last forecasts (β = 0.098) into account to carefully revise their expectations, which contributed to their better performance in price efficiency. The fact that bubbles in the sham and right anodal/left cathodal stimulation groups were larger than those in the left anodal/right cathodal stimulation group may be partly attributed to the differences between their forecasting rules.

**Table 3:** Estimated coefficients in the forecasting rules models.

*forecast_t_*	(1) left +/right -	(2) sham	(3) right +/left -
*price*_*t*-1_	0.909***	1.061***	1.001***
	(0.061)	(0.052)	(0.046)
*forecast*_*t*-1_	0.098***	-0.027	-0.018
	(0.0214)	(0.022)	(0.022)
*price*_*t*-1_ - *price*_*t*-2_	0.779***	1.138***	0.748***
	(0.019)	(0.188)	(0.168)
constant	–0.572	–2.239	1.71
	(0.382)	(3.623)	(2.968)

*Standard errors in parentheses. ^∗∗∗^*p* < 0.01*.

## Discussion

In this study, we examined the relationship between the DLPFC and asset bubble formation and discussed the cognitive mechanism behind it. Our studies are good supplements to asset bubble research. Previous studies have indicated that the DLPFC was activated during stock trading and the market bubble period (Ogawa et al., 2014; Raggetti et al., 2017). However, they did not discuss the causal relationship and transmission mechanism of the DLPFC and asset bubbles. According to the existing literature on asset bubbles, we found that many studies indicated that cognitive ability played an important role in asset trading and that a market consisting of participants with higher cognitive ability would generate fewer asset bubbles (Zong et al., 2017). Therefore, we inferred that cognitive ability might be an important transmission mechanism in reducing asset bubbles. Furthermore, we found that the DLPFC was closely related to cognitive processing (Nitsche et al., 2008; Hoy et al., 2013; Dedoncker et al., 2016). Therefore, we used tDCS to modulate the activity of the DLPFC and compared the asset bubbles and synchronous cognition improvements across different stimulation groups.

Our main concern was the influence of tDCS to the DLPFC on asset bubble formation. This problem has been rarely mentioned in previous studies about asset bubbles, which was our main contribution to asset bubble research. We would like to discuss the neural basis of asset bubbles and enrich the research on asset bubbles in the neuroscience field. In the classical learning-to-forecast experiment, the bubble of the left anodal/right cathodal group was significantly smaller than the bubble of the sham stimulation group. However, the right anodal/left cathodal group and sham group were not significantly different from each other.

We also examined the cognition mechanism of the effect of DLPFC stimulation on asset bubbles. Therefore, we described the changes in cognitive ability pre- and post stimulation. From the results of the 2-back task, we arrived at the same conclusion as previous studies: anodal tDCS to the left DLPFC improved cognition. Anodal tDCS over the left DLPFC has been reported by many studies to increase subjects’ cognitive ability, especially working memory as measured by n-back tasks (Nitsche et al., 2008; Hoy and Fitzgerald, 2010; Zaehle et al., 2011). In our experiment, we asked all subjects to complete a 2-back task before and after stimulation. We found that the accuracy of the 2-back task was significantly improved in subjects receiving left anodal/right cathodal stimulation, while sham stimulation and right anodal/left cathodal stimulation had no influence on the accuracy. However, the decrease in reaction time was not significant in any group. It was possibly due to the fact that in our experiment, the reaction time was so short that we were not able to capture the change of it. Overall, these results demonstrated that anodal tDCS to the left DLPFC indeed improved subjects’ cognitive abilities.

Combining the two results described above, one possible explanation of this phenomenon is that anodal tDCS over the left DLPFC reduced asset bubbles by improving subjects’ cognitive abilities, to some extent contributing to a causal relationship between asset bubbles and cognitive ability. To examine whether cognitive abilities played a role in reducing asset bubbles, we considered a correlation analysis between the average 2-back accuracy and RAD that we used to measure bubbles. We found a negative correlation between them and the correlation coefficient equaled to -0.332. This result indicated that if one market consisted of subjects with higher average accuracy in the 2-back task, there would be fewer market bubbles. Although there was a smaller bubble in the left anodal/right cathodal group, asset bubbles generally existed in all groups. We explained this phenomenon with the results of the regression from the prediction-biased model. From those results, we noted that all groups overestimated the change in market prices with the sham and right anodal/left cathodal groups making more excessive overestimates than the left anodal/right cathodal group. This finding may explain why asset bubbles formed in all groups and why the bubble of the left anodal/right cathodal was smaller than the others. Our results were consistent with previous studies reporting that both high cognitive ability groups and low cognitive ability groups overestimated the market price, while the high cognitive ability groups made predictions closer to an unbiased estimation (Zong et al., 2017).

We discussed the correlation of 2-back task and learning-to-forecast experiment. Our results showed that the correlation coefficient between the average 2-back accuracy and RAD was -0.332 (Pearson correlation analysis, *p* = 0.179). If one market consisted of subjects with higher average accuracy in the 2-back task, there would be fewer market bubbles. However, the *p*-value showed that the negative relationship between subjects’ cognitive abilities and asset bubbles was not significant. We measured asset bubbles using RAD which was a market level data and thus we only had 18 observations in the correlation analysis. That could be a reason why the correlation was not significant. Then we used another way to investigate their relationship. We ranked all markets by subjects’ average accuracy in the 2-back task and divided them into two groups. And we found that the RAD of high cognition group was significantly smaller than that of low group. Combined with these results and our previous analysis, we concluded that stimulating the DLPFC decreased the asset bubble and the main reason could be attributed to the increase of cognitive ability.

We also discussed the differences in the behavioral rules of different groups (left anodal/right cathodal, sham, and right anodal/left cathodal). These results helped us to understand how the subjects forecasted asset price and why they produced different magnitudes of asset bubbles. We obtained a similar conclusion as previous studies. Previous studies indicated that a pure trend-following strategy might expand the volatility of the market price and finally induce a larger market bubble (Bao et al., 2017). Our results showed that the left anodal/right cathodal stimulation group used both adaptive expectation and trend-following rules while predicting prices. However, the sham stimulation group only applied trend-following rules. They failed to consider their former forecast price and only made decisions based on the trend in the market price. The differences in behavioral rules also explained why bubbles in the different groups were significantly different.

Our results could have some implications for financial markets in the real world. According to our results, regulatory authorities of the financial market should make more effort to improve the overall financial literacy of market participants. The higher financial literacy, the less irrational behaviors and the fewer bubbles in the markets. For example, it should be emphasized that the trend-following rule is a short-sighted strategy. Investors should focus on stocks’ long-term performances instead of using the trend-following rule frequently. In addition, the left anodal/right cathodal DLPFC stimulation has a positive effect on enhancing cognitive abilities and may be considered in therapeutic applications.

The limitation of our experiment was that the design of the market was relatively simple with only a forecasting task instead of including a trading asset task, which is more complex. In addition, we only considered working memory among the many components of cognitive functioning. These are all feasible extensions for future research to make the results more robust.

In conclusion, we found that left anodal/right cathodal tDCS over the DLPFC decreased the asset bubble, possibly due to the improvement of subjects’ cognitive abilities, while sham tDCS or right anodal/left cathodal tDCS had no significant influence.

## Ethics Statement

This study was carried out in accordance with the recommendations of Ethic Committee of Zhejiang University with written informed consent from all subjects. All subjects gave written informed consent in accordance with the Declaration of Helsinki. The protocol was approved by the Ethic Committee of Zhejiang University.

## Author Contributions

CC and XZ performed the experiments and drew the figures. XY and CC analyzed the data. XY, CC, XZ, and XJ wrote and revised the manuscript, designed the experiments, and approved the final version to be published.

## Conflict of Interest Statement

The authors declare that the research was conducted in the absence of any commercial or financial relationships that could be construed as a potential conflict of interest.

## Footnotes

1 One-way ANOVA test result showed that there was no significant difference in age for subjects in these three different groups [*F*(2,123) = 1.68, *p* = 0.1915]. Chi-square test results also showed that there were no significant differences in gender, major and education for subjects in these three groups [Pearson Chi2(2) = 5.14, *p* = 0.08; Pearson Chi2(2) = 1.01, *p* = 0.6; Pearson Chi2(2) = 0.85, *p* = 0.65, respectively].

2 All of our data could be find in Supplementary Material Tables 1–4.
