# Supplementary material for: Stimulating the Dorsolateral Prefrontal Cortex Decreases the Asset Bubble: A tDCS Study
Source: Front Psychol. 2019 May 9;10:1031. doi: 10.3389/fpsyg.2019.01031 (PMC6521735; doi:10.3389/fpsyg.2019.01031)
Supplement: Supplementary file 2 [file Table_2.docx]

**Instructions for Experiment Ⅰ**

A series of random letters A to Z were presented consecutively on the center of the computer screen for three minutes. Your task is to remember and respond to with a button press when the present letter is the same as the letter presented 2 trials earlier. Otherwise, no special action is needed. For example, if B, A, C, A is consecutively presented on the screen, you have to press the button at the time when the second A comes out. If A, B, C, A is consecutively presented on the screen, then you don’t have to press the button because the second A is not same as the letter B presented 2 trials earlier. The letter is randomly presented and each letter is presented for only two seconds. You are considered to remember correctly and win 1 point if the following two conditions are met.

1. If you press the button when the present letter is the same as the letter presented 2 trials earlier;
2. Or if you don’t respond with a button press when the present letter is different as the letter presented 2 trials earlier;

Once you satisfy either of the above two conditions, you will win 1 point.

Finally, your payoff in this task will totally depends on the points you win. 20 points equal to 1 yuan. We will announce your payoff until the end of experiment Ⅱ.
