# Supplementary material for: Stimulating the Dorsolateral Prefrontal Cortex Decreases the Asset Bubble: A tDCS Study
Source: Front Psychol. 2019 May 9;10:1031. doi: 10.3389/fpsyg.2019.01031 (PMC6521735; doi:10.3389/fpsyg.2019.01031)
Supplement: Supplementary file 3 [file Table_3.docx]

**Instructions for Experiment Ⅱ**

In this experiment you participate in a financial market. Your role in the market is a professional financial advisor for an investment firm. All you have to do is to make a prediction of the market price of a particular asset. Then the firm will decide on the quantity of the asset they should buy or sell totally based on your prediction. That is to say: your forecast is the only information the firm has on the future market price. Usually, higher price predictions make a firm to buy more or sell less and vice versa. The asset pays a fixed dividend of 3.3 yuan in the end of each period and treasury bill rate is 5% at the same time. There is a total of 50 periods in this experiment. In each period, you need to predict the price one period ahead, i.e. in period t, you need predict the market price of period t+1. Your payoff in this experiment only depends on the accuracy of your prediction, which is calculated as the distance between your prediction and the actual market price. The more accurate your prediction is, the higher payoff will you receive. A specific Payoff Table is given at the end of the instructions.

**About the price determination**

The price is determined by the following price adjustment rule: when there is more

demand (firm's willingness to buy) of the asset, the price goes up; when there is

more supply (firm's willingness to sell), the price will go down. There are several investment firms in this market and each of them is advised by a financial advisor like you. Generally, higher price you predict, more willingly will the firm buy more or sell less, which increases the demand and vice versa. Total demand and supply is largely determined by the sum of the individual demand of these firms.

**About your job**

Your only task in this experiment is to predict the market price in each time period as accurately as possible. Your prediction in period 1 should lie between 0 and 100. At the beginning of the experiment you are asked to give a prediction for the price in period 1. When all forecasters have submitted their predictions for the first period, the firms will determine the quantity to demand, and the market price for period 1 will be determined and made public to all forecasters. Based on the accuracy of your prediction in period 1, your earnings will be calculated. Subsequently, you are asked to enter your prediction for period 2. When all participants have submitted their prediction and demand decisions for the second period, the market price for that period, will be made public and your earnings will be calculated, and so on, for all 50 consecutive periods. The information you can refer to at period t (except the first period) consists of all past prices, your predictions and earnings.

Please note that due to liquidity constraint, your firm can only buy and sell up to a maximum amount of assets in each period. This means although you can submit any prediction for period 2 and all periods after period 2, if the price in last period is $p_{t-1}$ and your prediction is $p_{t}^{e}$, then the firm's trading decision is constrained by $p_{t}^{e}\in\left[ p_{t-1}-30,p_{t-1}+30 \right]$. More precisely, the firm will trade as if $p_{t}^{e}=p_{t-1}+30$ if $p_{t}^{e}>p_{t-1}+30$ and trade as if $p_{t}^{e}=p_{t-1}-30$ if $p_{t}^{e}<p_{t-1}-30$.

**About your payoff**

Your earnings depend only on the accuracy of your predictions. The earnings shown on the computer screen will be in terms of points. If your prediction is $p_{t}^{e}$ and the price turns out to be $p_{t}$ in period t, your earnings are determined by the following equation:

$$Payoff=max\left[ 1300-\frac{1300}{49}\left( p_{t}^{e}-p_{t} \right)^{2},0 \right]$$

The maximum possible points you can earn for each period (if you make no prediction

error) is 1300, and the larger your prediction error is, the fewer points you can make. You will earn 0 points if your prediction error is larger than 7. There is a Payoff Table below for reference, which shows the points you can earn for different prediction errors.

We will pay you in cash at the end of the experiment based on the points you earned. You earn 1 yuan for each 2000 points you make.

Payoff Table

| Payoff Table for Forecasting Task  $Your Payoff=max\left[ 1300-\frac{1300}{49}\left( Your Prediction Error \right)^{2},0 \right]$  2000 points equal 1 yuan | | | | | | | |
| --- | --- | --- | --- | --- | --- | --- | --- |
| error | points | error | points | error | points | error | points |
| 0.00 | 1300 | 1.85 | 1209 | 3.70 | 937 | 5.55 | 483 |
| 0.05 | 1300 | 1.90 | 1204 | 3.75 | 927 | 5.60 | 468 |
| 0.10 | 1300 | 1.95 | 1199 | 3.80 | 917 | 5.65 | 453 |
| 0.15 | 1299 | 2.00 | 1194 | 3.85 | 907 | 5.70 | 438 |
| 0.20 | 1299 | 2.05 | 1189 | 3.90 | 896 | 5.75 | 423 |
| 0.25 | 1298 | 2.10 | 1183 | 3.95 | 886 | 5.80 | 408 |
| 0.30 | 1298 | 2.15 | 1177 | 4.00 | 876 | 5.85 | 392 |
| 0.35 | 1297 | 2.20 | 1172 | 4.05 | 865 | 5.90 | 376 |
| 0.40 | 1296 | 2.25 | 1166 | 4.10 | 854 | 5.95 | 361 |
| 0.45 | 1295 | 2.30 | 1160 | 4.15 | 843 | 6.00 | 345 |
| 0.50 | 1293 | 2.35 | 1153 | 4.20 | 832 | 6.05 | 329 |
| 0.55 | 1292 | 2.40 | 1147 | 4.25 | 821 | 6.10 | 313 |
| 0.60 | 1290 | 2.45 | 1141 | 4.30 | 809 | 6.15 | 297 |
| 0.65 | 1289 | 2.50 | 1134 | 4.35 | 798 | 6.20 | 280 |
| 0.70 | 1287 | 2.55 | 1127 | 4.40 | 786 | 6.25 | 264 |
| 0.75 | 1285 | 2.60 | 1121 | 4.45 | 775 | 6.30 | 247 |
| 0.80 | 1283 | 2.65 | 1114 | 4.50 | 763 | 6.35 | 230 |
| 0.85 | 1281 | 2.70 | 1107 | 4.55 | 751 | 6.40 | 213 |
| 0.90 | 1279 | 2.75 | 1099 | 4.60 | 739 | 6.45 | 196 |
| 0.95 | 1276 | 2.80 | 1092 | 4.65 | 726 | 6.50 | 179 |
| 1.00 | 1273 | 2.85 | 1085 | 4.70 | 714 | 6.55 | 162 |
| 1.05 | 1271 | 2.90 | 1077 | 4.75 | 701 | 6.60 | 144 |
| 1.10 | 1268 | 2.95 | 1069 | 4.80 | 689 | 6.65 | 127 |
| 1.15 | 1265 | 3.00 | 1061 | 4.85 | 676 | 6.70 | 109 |
| 1.20 | 1262 | 3.05 | 1053 | 4.90 | 663 | 6.75 | 91 |
| 1.25 | 1259 | 3.10 | 1045 | 4.95 | 650 | 6.80 | 73 |
| 1.30 | 1255 | 3.15 | 1037 | 5.00 | 637 | 6.85 | 55 |
| 1.35 | 1252 | 3.20 | 1028 | 5.05 | 623 | 6.90 | 37 |
| 1.40 | 1248 | 3.25 | 1020 | 5.10 | 610 | 6.95 | 19 |
| 1.45 | 1244 | 3.30 | 1011 | 5.15 | 596 | $\geq$7.00 | 0 |
| 1.50 | 1240 | 3.35 | 1002 | 5.20 | 583 |  |  |
| 1.55 | 1236 | 3.40 | 993 | 5.25 | 569 |  |  |
| 1.60 | 1232 | 3.45 | 984 | 5.30 | 555 |  |  |
| 1.65 | 1228 | 3.50 | 975 | 5.35 | 541 |  |  |
| 1.70 | 1223 | 3.55 | 966 | 5.40 | 526 |  |  |
| 1.75 | 1219 | 3.60 | 956 | 5.45 | 512 |  |  |
| 1.80 | 1214 | 3.65 | 947 | 5.50 | 497 |  |  |
