# Supplementary material for: Stimulating the Dorsolateral Prefrontal Cortex Decreases the Asset Bubble: A tDCS Study
Source: Front Psychol. 2019 May 9;10:1031. doi: 10.3389/fpsyg.2019.01031 (PMC6521735; doi:10.3389/fpsyg.2019.01031)
Supplement: Supplementary file 4 [file Table_4.DOCX]

Appendix

Stage1: 2-back task

C

A

B

A

E

……

In this stage, participants would see a series of random letters presented consecutively on the screen for 3 minutes. They needed to remember the order of the letters and respond by pressing the SPACE button when the present letter was the same as the letter presented 2 trials earlier. The 2-back task as a whole consists of 90 trials containing approximately 25% targets. Each letter was presented for 2 s in a trial. All participants were asked to perform a 2-back task before and immediately after stimulation, and the order of stimulus presentations were exactly the same across different groups.

Stage2: learning-to-forecast experiment

Period:2

Your forecast: 61

Your history forecast: 59

Your Final Payment:

45.6 RMB

Period:50

Your forecast: 59

Your history forecast:

......

…

Market Price: 60

Your forecast: 59

Payoff: 1273.5

Period:1

Your forecast: 59

……

In this stage, subjects had to forecast the asset price in every period, after they finished their prediction we would show them the results of this period (market price and their payoff in this period). Then they needed to start next period’s prediction. In each period, historical prices were displayed to the subjects for reference. The whole experiment lasted 50 rounds. When subjects finished 50 rounds prediction, they would see their final payment in the screen.
